
JOURNAL INFORMATION
==============================
NLM Title Abbreviation: JBRA Assist Reprod
Journal ID: JBRA Assist Reprod
NLM Journal ID: 101684552
Journal ID: jbra

JBRA Assisted Reproduction

ISSN: 1517-5693
EISSN: 1518-0557
Publisher: Brazilian Society of Assisted Reproduction

ARTICLE INFORMATION
==============================
PMCID: PMC5473708
PMID: 28609282
DOI: 10.5935/1518-0557.20170030
Article version: 1
Subjects: Oral Presentations

Oral Presentations: Abstracts of the 13th Red Lara Taller General, Buenos Aires, Argentina, 26-28 April 2017

Publication date: 2017 Apr-Jun
Print publication date: 2017 Apr-Jun
Volume: 21
Issue: 2
First page: 137
Last page: 141
License: This is an Open Access article distributed under the terms of the Creative Commons Attribution License, which permits unrestricted use, distribution, and reproduction in any medium, provided the original work is properly cited.
License URL: https://creativecommons.org/licenses/by/4.0/

==============================


O-O1. Zika Virus Outbreak - Assisted reproduction patients should avoid pregnancy?

Borges Jr. E. 1 2
Braga D.P.A.F. 1 3
Zanetti B.F. 1
Setti A.S. 2
Provenza R.R. 1
Iaconelli Jr. A. 1
1 Fertility - Medical Group, São Paulo, SP - Brazil
2 Instituto Sapientiae - Centro de Estudos e Pesquisa em Reprodução Humana Assistida, São Paulo, SP - Brazil
3 Disciplina de Urologia, Área de Reprodução Humana, Departamento de Cirurgia, Universidade Federal de São Paulo. - UNIFESP

O-02. FSH dose to stimulate different patient' ages: when less is more

Borges Jr. E. 1 2
Zanetti B.F. 2
Setti A.S. 2
Braga D.P.A.F. 1 3
Figueira R.C.S. 1
Iaconelli Jr. A. 1
1 Fertility - Medical Group, São Paulo, SP - Brazil
2 Instituto Sapientiae - Centro de Estudos e Pesquisa em Reprodução Humana Assistida, São Paulo, SP - Brazil
3 Disciplina de Urologia, Área de Reprodução Humana, Departamento de Cirurgia, Universidade Federal de São Paulo. - UNIFESP

O-03. The adverse effect of overweight in Assisted Reproduction Outcomes

Sampo A.V. 1
Palena C. 1
Ganzer L. 1
Maccari V. 1
Estofán G. 1
Hernández M. 1
1 CIGOR - Centro Integral de Ginecología, Obstetricia y Reproducción. Córdoba, Argentina

O-04. Can ovarian doublestimulation in the same menstrual cycle improve the results of IVF?

Cardoso M.C.A. 1
Evangelista A. 1 2
Sartório C. 1
Vaz G. 1 2
Werneck C.L.V. 1
Guimarães F.M. 1
Sá P.G. de 1 2
Erthal M.C. 1
1 Vida - Centro de Fertilidade, Rio de Janeiro, Brazil
2 Department of Gynecology of the State University of Rio de Janeiro (UERJ)

O-05. Key performance indicators score (KPIs-score) based on clinical and laboratorial parameters can establish benchmarks for internal quality control in an ART program

Franco Jr J.G. 1 2
Petersen C.G. 1 2
Mauri A.L. 1 2
Vagnini L.D. 2
Renzi A. 2
Petersen B. 2
Mattila M.C. 1
Comar V.A. 1
Ricci J. 1
Dieamant F. 1 2
Oliveira J.B.A. 1 2
Baruffi R.L.R. 1 2
1 Center for Human Reproduction Prof. Franco Jr., Ribeirao Preto, Brazil
2 Paulista Center for Diagnosis, Research and Training, Ribeirao Preto, Brazil

O-06. Morphological embryo selection: An elective Single Embryo Transfer proposal

Déniz F.P. 1
Encinas C. 1
Fuente J. La 1
1 Embriovid, La Paz, Bolivia

O-07. Blastocyst classification systems used in Latin America: is a consensus possible?

Puga-Torres T. 1
Blum-Rojas X. 1
Blum-Narváez M. 1
1 Centro Nacional de Reproducciónn Asistida INNAIFEST - Guayaquil - Ecuador

O-08. Influence of the abstinence period on human sperm quality: analysis of 2,458 semen samples

Comar V.A. 1
Petersen C.G. 1 2
Mauri A.L. 1 2
Mattila M. 1
Vagnini L.D. 2
Renzi A. 2
Petersen B. 2
Nicoletti A. 1
Dieamant F. 1 2
Oliveira J.B.A. 1 2
Baruffi R.L. R. 1 2
Franco Jr. J.G. 1 2
1 Center for Human Reproduction Prof. Franco Jr., Ribeirao Preto, SP, Brazil
2 Paulista Center for Diagnosis Research and Training, Ribeirao Preto, SP, Brazil

O-09. Semen parameters in men with varicocele: DNA fragmentation, chromatin packaging, mitochondrial membrane potential, and apoptosis

Dieamant F. 1 2
Petersen C.G. 1 2
Mauri A.L. 1 2
Conmar V.A. 1
Matilla M.C. 1
Vagnini L.D. 2
Renzi A. 2
Petersen B. 2
Zamara C. 1
Oliveira J.B. A. 1 2
Baruffi R.L.R. 1 2
Franco Jr. J.G. 1 2
1 Centre for Human Reproduction Prof Franco Jr, Research, Ribeirao Preto, Brazil
2 Paulista Center for Diagnosis Research and Training, Research, Ribeirao Preto, Brazil

0-10. Effect of sperm DNA fragmentation on embryo development and biological aspects

Alvarez Sedó C. 1
Bilinski M. 1
Lorenzi D. 1
Uriondo H. 1
Noblía F 1
Longobucco V. 1
Lagar E. Ventimiglia 1
Nodar F. 1
1 Centro de Estudios en Genética y Reproducción (CEGYR), Buenos Aires, Argentina

O-11. Impact of the Z potential technique on the reduction of sperm DNA fragmentation index, fertilization rate and embryo development

Duarte C. 1
Núñez V. 1
Wong Y. 1 2
Vivar C. 1
Benites E. 1
Rodriguez U. 1
Vergara C. 1
Ponce J. 1
1 NIU VIDA Specialized Center for Assisted Reproduction, Lima, Peru
2 Laboratory of Animal Biotechnology, Ricardo Palma University, Lima, Peru

O-12. A healthy HLA matching baby born by using a combination of aCGH and Karyomapping: The first Latin America case

Delgado-Elías A. 1 2
Llerena G. 1
López R. 3
Portella J. 1
Inoue N. 1
Noriega-Hoces L. 1 2
Guzman L. 1 3
1 PRANOR Laboratorio. Grupo de Reproducción Asistida. San Isidro. Lima. Peru
2 Clinica Concebir. San Isidro. Lima. Peru
3 Reprogenetics Latinoamérica, Lima-Peru

O-13. Expanded carrier screening in gamete donors of Venezuela

Urbina M.T. 1
Benjamin I. 1
Medina R. 1
Jiménez J. 1
Trías L. 1
Lerner J. 1
1 Unifertes Fertility Unit, Caracas, Venezuela

Soledad Sepúlveda Award

Best Oral Presentation.

13º REDLARA General Congress – Buenos Aires – Argentina 2017

